# Diversity of Oligopeptide Transport in Yeast and Its Impact on Adaptation to Winemaking Conditions

**DOI:** 10.3389/fgene.2020.00602

**Published:** 2020-06-10

**Authors:** Carmen Becerra-Rodríguez, Souhir Marsit, Virginie Galeote

**Affiliations:** ^1^SPO, INRAE, Université de Montpellier, Montpellier SupAgro, Montpellier, France; ^2^Institut de Biologie Intégrative et des Systèmes, Regroupement Québécois de Recherche sur la Fonction, l’Ingénierie et les Applications des Protéines, (PROTEO), Département de Biologie, Université Laval, Québec City, QC, Canada

**Keywords:** oligopeptides, peptide transport, yeast, wine fermentation, *Saccharomyces cerevisiae*, Fungal Oligopeptide Transporters, adaptation

## Abstract

Nitrogen is an essential nutrient for yeasts and its relative abundance is an important modulator of fermentation kinetics. The main sources of nitrogen in food are ammonium and free amino acids, however, secondary sources such as oligopeptides are also important contributors to the nitrogen supply. In yeast, oligopeptide uptake is driven by different families of proton–coupled transporters whose specificity depends on peptide length. Proton-dependent Oligopeptide Transporters (POT) are specific to di- and tri-peptides, whereas the Oligopeptide Transport (OPT) family members import tetra- and pentapeptides. Recently, the novel family of Fungal Oligopeptide Transporters (FOT) has been identified in *Saccharomyces cerevisiae* wine strains as a result of a horizontal gene transfer from *Torulaspora microellipsoides*. In natural grape must fermentations with *S. cerevisiae*, Fots have a broader range of oligopeptide utilization in comparison with non-Fot strains, leading to higher biomass production and better fermentation efficiency. In this review we present the current knowledge on the diversity of oligopeptide transporters in yeast, also discussing how the consumption of oligopeptides provides an adaptive advantage to yeasts within the wine environment.

## Introduction

Yeasts are unicellular, eukaryotic organisms that are commonly found in food environments, where they normally consume sugars to produce energy in anaerobic conditions. This process is known as fermentation, a reaction by which carbohydrates and other organic molecules are catabolized to release energy in the absence of oxygen, using an organic molecule as the final electron acceptor. Alcoholic fermentation, i.e., the transformation of monomeric sugars into ethanol and carbon dioxide, is the predominant form of fermentation in yeast. Several foods and beverages such as bread, sake, beer or wine are the result of such alcoholic fermentation performed by yeasts and other microorganisms.

As any other known form of life, yeasts require nitrogen for building some essential macromolecules, i.e., proteins and nucleic acids. This makes nitrogen one of the main nutrients and therefore a key modulator in yeast life cycle. In a winemaking context, nitrogen availability controls yeast growth rate and fermentation kinetics, nitrogen deficiency being the first cause of slow and incomplete fermentations (Alexandre and Charpentier, 1998; Bisson, 1999; Bell and Henschke, 2005). Indeed, nitrogen supplementation during fermentation effectively prevents sluggish or stuck fermentations (Blateyron and Sablayrolles, 2001). Nitrogen availability also affects the formation of volatile compounds during fermentation, which are largely responsible for shaping wine aroma profiles and thus are an important enological trait (Hazelwood et al., 2008). Ammonium and free amino acids are the primary nitrogen sources for yeast, as main constituents of total assimilable nitrogen in grape must (Henschke and Jiranek, 1993). However, there are other molecules, such as oligopeptides, polypeptides, proteins, amides, biogenic amines and even nucleic acids that constitute secondary and diverse sources of nitrogen (Ough et al., 1991).

Oligopeptides, also simply termed peptides, consist in short sequences of a few amino acids covalently joined by peptide bonds (Nelson and Cox, 2013). In nature, they mainly result from proteolytic processes. Analysis of the nitrogen sources in soils established that oligopeptides correspond to the peptide fraction with a molecular weight < 1 kDa, which is equivalent to peptides with 2–9 amino acid residues (Farrell et al., 2011). Oligopeptide composition in foods and beverages is highly diverse, since it depends on the industrial and/or domestic processing that these products usually undergo during manufacturing (Clapperton, 1971; Henschke and Jiranek, 1993; Takahashi et al., 2012; D’Souza et al., 2018). In grape must, peptide composition is also conditioned by the grape variety, terroir or vintage, among other environmental factors (Moreno-Arribas et al., 1996). However, some amino acids have been found as predominant in the peptide fractions of different samples. Previous studies on Chardonnay grape must from Coursan (France, 2012) showed that glutamate and glutamine were the most abundant amino acids in the oligopeptide fraction, followed by glycine, asparagine and aspartate (Marsit et al., 2015); a similar composition was found in the peptide fractions of Koshu wines, beers, or soy (Yokotsuka et al., 1975; Dale and Young, 1989; Kitagawa et al., 2008). Nevertheless, analyses of oligopeptide composition in grape musts and other foods and beverages are still scarce.

In the same way as bacteria, plants, mammals and other fungi, yeasts can uptake oligopeptides from the environment for use as nitrogen and carbon sources. In grape must, the oligopeptide fraction constitutes 17% of total nitrogen and 10% of the nitrogen assimilated by yeast (Yokotsuka and Fukui, 2002; Marsit et al., 2015). In beer, 30–50% of wort peptides are utilized during fermentation as nutrients and precursors of the cell wall, although growth rate is lower when oligopeptides are the sole source of nitrogen (Clapperton, 1971; Ingledew and Patterson, 1999; Mo et al., 2013). Furthermore, Mo et al. (2013) showed that only peptide fractions below 5 kDa (corresponding to peptides with up to 45 amino acid residues approximatively) were assimilated by yeast during beer fermentation, with a particularly fast decrease in peptides below 1 kDa. Extracellular protease activity is highly regulated, allowing yeasts to degrade the polypeptides from wort into assimilable oligopeptides (Lekkas et al., 2009). While the uptake of some amino acids may be strictly inhibited or repressed, yeasts can indirectly acquire those amino acids through the consumption of oligopeptides and their subsequent hydrolysis (Kevvai et al., 2016; Marsit et al., 2016). In wine fermentation, oligopeptides assimilation can lead to the release of some amino acids that are then consumed after depletion of the other nitrogen sources (Marsit et al., 2016). Furthermore, experiments on synthetic media containing ammonia and free amino acids supplemented with yeast hydrolysate have shown that nitrogen provided by peptides eventually constitutes 40% of the yeast protein fraction during alcoholic fermentation, which highlights the important anabolic role of oligopeptides versus other nitrogen sources such as ammonium, which only contributes 20% of this fraction (Kevvai et al., 2016).

Besides being a nutrient source, some oligopeptides have other important, specific functions within living organisms and environments. Glutathione (γ-L-Glutamyl-L-cysteinylglycine) is a tripeptide whose antioxidant functions in the cell have been extensively described. In wine fermentation, glutathione from grape musts mitigates the oxidative stress caused by copper and prevents browning reactions by blocking the oxidation of brown pigment precursors (Rigaud et al., 1991; Zimdars et al., 2019). Small peptides found in foods are taste determinants of umami, along with glutamate and ribonucleotides (Zhang et al., 2017). Other peptides are produced by bacteria, fungi, plants and animals as antimicrobial agents, and thus some of them have been commercially used as antibiotics and bioactive compounds in food (Rai and Jeyaram, 2015; Yang and Yousef, 2018). In addition, some of the described antifungal peptides have other reported activities within cells, such as antioxidants, protease and ion channel inhibitors or surface-immobilized peptides (Wang et al., 2016; see The Antimicrobial Peptide Database)^1^. In yeast, killer toxins constitute a widespread phenotype of peptides that are produced to penetrate and kill cells through interaction with membrane-associated complex carbohydrates, usually targeting spoilage and pathogenic microorganisms in fermentative environments (Mannazzu et al., 2019). More precisely, it has been described how some *Saccharomyces cerevisiae* strains secrete peptides to inhibit the growth of non-*Saccharomyces* yeasts during fermentation, enabling competitors exclusion and broader access to nutrients (Albergaria et al., 2010; Branco et al., 2017).

Despite the importance that oligopeptides seem to have in yeast performance during fermentation, their role has been long underestimated, which explains the insufficient number of studies dedicated to this issue. Here we provide a state-of-the-art review about oligopeptide transport in yeast, focusing on the impact of peptide uptake during wine fermentation and the role of oligopeptide transporters in the adaptation of *S. cerevisiae* to wine environments.

## Families of Oligopeptide Transporters in Yeast

Oligopeptide transport is a mechanism that has been retained throughout evolution, as demonstrated by its presence in bacteria, fungi, plants and mammals. It consists in the energy-dependent uptake of small peptides across the biological membrane into the cell. It works as an independent process from amino acid transport, with high stereoselectivity for α-peptide bonds and amino acid residues in L-form in all transport systems (Steiner et al., 1995). In bacteria, peptide transport is mainly carried out by the ATP-binding cassette (ABC) superfamily, with Dpp and Opp as the best-known systems (Higgins and Gibson, 1986; Manson et al., 1986; Hiles et al., 1987). ABC transporters are characterized by two transmembrane domains and two nucleotide-binding domains that enable peptide import through the hydrolysis of two ATP molecules (reviewed by Rees et al., 2009). Although the ABC superfamily is widespread among eukaryotes, some of its members have evolved into functionalities different from nutrient uptake. For example, ABC transporters in entomopathogenic fungi mediate host-pathogen interactions, working as metabolite efflux pumps (Baral, 2017). In *S. cerevisiae*, ABC transporters are involved in peptide pheromone secretion, regulation of mitochondrial function, vacuolar detoxification, pleiotropic drug resistance and stress adaptation (Jungwirth and Kuchler, 2006). In fungi, peptide uptake is generally energized by the symport with protons, depending thus on proton-coupled transporters. It is noteworthy that peptide transport is not an essential mechanism, since mutation-induced inactivation of transport systems has been proved not to be lethal (Perry et al., 1994). Nevertheless, the development of multiple transport systems (as illustrated in Table 1) among fungi and yeasts illustrates the importance of oligopeptide transport to cope with different nitrogen sources for survival.

**TABLE 1:** List of oligopeptide transport systems.

Family of peptide transporters	Organisms	Energy source for transport	Examples	Species	Substrates
ABC	Bacteria	ATP hydrolysis	Dpp, Opp	*Escherichia coli Salmonella typhimurium Streptococcus pneumoniae*	Dipeptides, Oligopeptides
POT/PTR	Bacteria, fungi, mammals, plants	Proton-coupled symport	Ptr2	*S. cerevisiae C. albicans A. thaliana*	Di- and tripeptides
			DtpT	*L. lactis*	
			PepT	*S. oneidensis S. thermophilus*	
			PepT1, PepT2	*Homo sapiens*	Di- and tripeptides, β-lactam antibiotics, antiviral and anticancer drugs
OPT	Bacteria, fungi, mammals, plants	Proton-coupled symport	Opt1	*S. cerevisiae C. albicans S. pombe*	Tetra- and pentapeptides, glutathione, enkephalins
			Opt2	*S. cerevisiae C. albicans*	Tetrapeptides
			Opt3, Opt4, Opt5, Opt6, Opt7, Opt8	*C. albicans*	Up to eight amino acid residues
			Ys1	*A. thaliana Zea mays*	Peptides and metal-NA complexes
FOT	Fungi	Proton-coupled symport	Fot1*, Fot2*	*S. cerevisiae* wine strains	Di- and tripeptides, glutathione
			Fot3*		Non-characterized
			FotX, FotY, Fot2tomi	*T. microellipsoides*	Non-characterized
Dal5	Yeast	Cation/proton-coupled symport		*S. cerevisiae*	Allantoate, ureidosuccinate, dipeptides
Asp3	Yeast	Unknown		S. cerevisiae* W. anomalus	Dipeptides with asparagine in the C-terminal
Gap1	Yeast	Unknown		*S. cerevisiae*	γ-glutamyl dipeptides

*Originated from a HGT phenomenon. The table includes some examples of the families of transporters that have shown oligopeptide transport activity.

### Proton-Dependent Oligopeptide Transport Family

Paulsen and Skurray (1994) first designated the Proton-dependent Oligopeptide Transport (POT) family after finding sequence similarity between different non-ABC-type, proton-dependent transporters, such as PepT1 and PepT2 in mammals, *Arabidopsis thaliana* nitrate transporter Ckl1, DtpT oligopeptide transporter in *Lactococcus lactis* or the *S. cerevisiae* peptide transporter Ptr2. They predicted a shared topological domain consisting in 12 putative α-helical transmembrane segments, with two specific conserved motifs that had not been found in any other protein at that time. However, Steiner et al. (1995) considered that the designation POT was inappropriate, since not all the transporters in the denominated POT family had been proved to be proton-dependent or to mediate oligopeptide transport. They therefore proposed the use of the term Peptide Transport family (PTR) to refer to a group of non-ABC-type, proton-dependent oligopeptide transporters with a high similarity at protein sequence level and multiple conserved motifs for phosphorylation and glycosylation. Currently, the PTR or POT denominations are indiscriminately used in literature referring to the proton-dependent oligopeptide transporters family with specificity for di- and tri-peptides. POT have been identified in both eukaryotes and prokaryotes, with the exception of archaea. A possible explanation for this exception is the absence of peptides in the extreme environments usually inhabited by archaea (Newstead, 2017). The POT family has been grouped as a member of the Major Facilitator Superfamily (MFS) in the Transporter Classification Database (TCDB,^2^ reference for POT: TC# 2.A.17).

In *S. cerevisiae*, the PTR system consists of three interdependent genes: *PTR2* encodes the integral membrane transporter Ptr2, the best-known member of the PTR family with isoforms also found in *Candida albicans* (Basrai et al., 1992) and *A. thaliana* (Steiner et al., 1994). *PTR1* and *PTR3* work as regulatory elements: *PTR1* is essential for the expression of *PTR2* and consequently for transporter functionality; on the other hand, *PTR3* is involved in the induction of peptide transport in presence of poor sources of nitrogen, although its activity is not crucial for di- and tripeptide transport by Ptr2 (Barnes et al., 1998). Despite its broadly multi-specific substrate preferences, Ptr2 from *S. cerevisiae* has a higher affinity for di- and tri-peptides containing aromatic, branched or basic amino acids (Phe, Trp, Tyr, Ile, Leu, Val, Arg, Lys, and His) and a lower affinity for negatively charged amino acids (Asp and Glu), glycine and proline (Ito et al., 2013). Additionally, members of the POT family in plants have acquired the ability to transport other nitrogen sources, such as the nitrate permease AtCHL1 in *A. thaliana* (Steiner et al., 1995; Williams and Miller, 2001; Léran et al., 2014).

### Oligopeptide Transporters

All ATP-binding cassette and PTR have been found to transport only di- and tri-peptides (Becker and Naider, 1980; Ganapathy and Leibach, 1991; Payne and Smith, 1994). However, Lubkowitz et al. (1997) identified a new oligopeptide transporter in *C. albicans* able to uptake peptides with four or five amino acid residues with high affinity. This new transporter was independent of the di- and tripeptide transport systems, as shown by substrate competition experiments using radiolabeled peptides. The lack of sequence homology of this new transporter with the PTR or ABC transporters demonstrated that it belonged to a novel family of oligopeptide transporters, receiving the name of Opt1 from Oligo Peptide Transporter (OPT; TC# 2.A.67). In the following years, sequence homology analyses enabled the identification of new isoforms of the OPT family in *Schizosaccharomyces pombe* (Lubkowitz et al., 1998), Opt1 and Opt2 in *S. cerevisiae* (Hauser et al., 2000) and up to four new functional members in *C. albicans* (Opt2-3-4-5). Moreover, OPT transporters were also identified in prokaryotes and plants (Hauser et al., 2001; Gomolplitinant and Saier, 2011). Studies with the Opt1 isoform in *S. cerevisiae* showed that the OPT system also works with the proton-motive force (Hauser et al., 2001; Osawa et al., 2006). The deduced protein sequence of Opt1 in *C. albicans* revealed a membrane-associated, hydrophobic protein with 12 putative transmembrane domains (Wiles et al., 2006b). However, later studies revealed that OPT transporters actually contained 16 transmembrane domains originated from sequential duplication events of a 2-transmembrane domain precursor element (Gomolplitinant and Saier, 2011).

Although OPT are often defined as tetra- and pentapeptide transporters, some isoforms in *C. albicans* are able to uptake peptides of up to eight amino acid residues (Reuß and Morschhäuser, 2006). Opt1 can also transport glutathione, with higher affinity for the reduced form, as well as the pentapeptides known as enkephalins (Bourbouloux et al., 2000; Hauser et al., 2000; Zulkifli et al., 2016). The other OPT member in *S. cerevisiae*, Opt2, shares 31% identity with Opt1, and can transport tetrapeptides. Opt2 participates in the fusion of small vesicles into a larger vacuole, which plays an important role in drug detoxification processes (Aouida et al., 2009). Additionally, Opt2 is involved in the regulation of Opt1 expression (Hauser et al., 2001). The third putative member *OPT3* (also referred to as *YGL114w*) has only been identified through a low sequence homology, with no evidence of any encoded and expressed protein in yeast (Hauser et al., 2001; Wiles et al., 2006b). Regarding substrate specificity, there is still a lack of information related to the composition of the oligopeptides preferred by OPT transporters. However, it is known that OPT have a broad range of distinct substrates with peptide-length specificity (Osawa et al., 2006; Reuß and Morschhäuser, 2006).

### Fungal Oligopeptide Transporters

Using a complementary DNA expression library from an environmental sample of soil eukaryotes, Damon et al. (2011) identified six putative genes for di- and tripeptide transporters that were not homologous to Ptr2. The predicted protein structure determined 11 transmembrane domains and a N-terminal cytosolic tail. These six sequences shared high sequence identity, with homologous sequences found in other fungal species. When phylogenetic analyses were performed, the six putative transporter genes grouped with 15 other fungal sequences that belonged to 10 species from the Ascomycota and Basidiomycota phyla. Although none of these 15 sequences had been previously characterized, many of them had already been annotated as putative amino acid transporters. Due to the specificity of this new group to the fungi kingdom, they proposed the denomination Fungal Oligopeptide Transporters (FOT). Additionally, experiments in *Xenopus* oocytes demonstrated that the proton symport was the driving force of FOT peptide transport (Damon et al., 2011).

The phylogenetic analysis performed by Damon et al. (2011) identified two members of the *FOT* gene family in *S. cerevisiae* wine strain EC1118. The tandem *FOT1-2* genes in EC1118 had been previously annotated as permeases for neutral amino acids by Novo et al. (2009), who sequenced the strain’s genome. Marsit et al. (2015) reported that *FOT* genes were only present in *S. cerevisiae* wine strains as a result of a horizontal gene transfer (HGT) from the species *Torulaspora microellipsoides*. Sequence analysis enabled the identification of *FOT2* from EC1118 as an isoform of *FOT2_tomi* in *T. microellipsoides*, since they share the same sequence (Table 2A). Conversely, *FOT1* from EC1118 or *FOT3* from the wine strain K1 were the result of gene conversions between the two tandem genes *FOTX* and *FOT2_tomi* in *T. microellipsoides* (Marsit et al., 2015). In any case, all the identified FOT members share a high sequence homology at gene and protein level (Table 2B), with *FOTY* from *T. microellipsoides* as the most divergent.

**TABLE 2:**
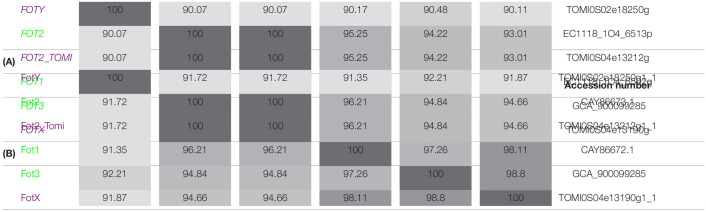
Fungal Oligopeptide Transporters share a high sequence identity at gene and protein level.

*Percentage identity matrices were built by multiple-alignments with the six FOT genes **(A)** and protein sequences **(B)** from S. cerevisiae (in green) and T. microellipsoides (in purple) using Clustal Omega (Sievers et al., 2011). The guide trees to the right show the distance between the sequences based on the pairwise scores. FOT1 and FOT2 protein and gene accession numbers correspond to the NCBI database; FOTX, FOTY and FOT2_TOMI accession numbers correspond to the Genome Resources for Yeast Chromosomes (GRYC) database; FOT3 sequence can be found in the genome assembly for S. cerevisiae strain K1-28-2B deposited at the European Nucleotide Archive (ENA).*

Currently, only few studies have been performed on FOT members in *S. cerevisiae*, with scarce information regarding those members in the non-*Saccharomyces* species. In 59A, a haploid derivative strain of EC1118, FOT transporters have shown a wide specificity for di- and tripeptides, particularly those containing glutamate (Damon et al., 2011; Marsit et al., 2015). The comparison of peptide consumption between strains with Fot1-2 and fot1-2 deleted mutants showed that the wild type could consume up to 118 dipeptides and 8 tripeptides, whereas the fot1-2 mutant could only consume 28 di-peptides and 4 tri-peptides (Damon et al., 2011). In addition, FOT-containing yeasts also consumed more glutathione (Marsit et al., 2015).

Some members of the FOT family may have evolved into different peptide substrate specificities, acquiring potentially specialized functions, however, this possibility still remains unexplored. Furthermore, FOT have only been characterized for di/tripeptide transport, whereas it is still not known whether they are also able to import tetra-, penta- or even deca-peptides.

### Other Permeases With Peptide Transport Activity

Other permeases with another main function in yeast have also shown peptide transporter activity. Dal5 is an allantoate/ureidosuccinate permease that is able to import dipeptides into the cytoplasm in *S. cerevisiae*. As a member of the allantoate permease family (TC# 2.A.1.14), it belongs to the anion:cation symporter subfamily from the MFS, although it is not known whether Dal5 works in symport with protons or another cation (Nelissen et al., 1997; Hellborg et al., 2008). Even though its affinity for allantoate, ureidosuccinate and allantoin is higher than that for peptides, Dal5 constitutes the predominant dipeptide transporter in some *S. cerevisiae* strains, such as the wild-type strain W303 (Cai et al., 2007). Contrary to Ptr2, Dal5 preferably transports non-N-end rule dipeptides, i.e., peptides without basic (Arg, His, Lys) or bulky hydrophobic (Ile, Leu, Phe, Trp, or Tyr) amino acid residues at the N terminus, which highlights the complementary activities of the different di- and tripeptide transporters (Cai et al., 2007). It has also been suggested that the amino acid permease asparaginase II Asp3p can transport dipeptides with asparagine in the C-terminal by an as-yet-unidentified mechanism (Homann et al., 2005). Interestingly, the presence of the *ASP3* locus in *S. cerevisiae* seems to be due to a horizontal gene transfer phenomenon from the wine yeast *Wickerhamomyces anomalus* (League et al., 2012). However, *ASP3* locus is not homogeneous among *S. cerevisiae* strains; for example, a functional *ASP3* gene is absent from wine strain EC1118, vineyard isolate Y55 or different clinical isolates (Lashkari et al., 1997; Homann et al., 2005; Novo et al., 2009). Additionally, it has been reported that general amino acid permease Gap1 is responsible for the consumption of γ-glutamyl dipeptides when they were the sole nitrogen sources in the medium, with no apparent implication of Ptr2, Dal5 or Opt1-2 (Rubio-Texeira et al., 2012).

## Regulation of Oligopeptide Transport

**FIGURE 1:**
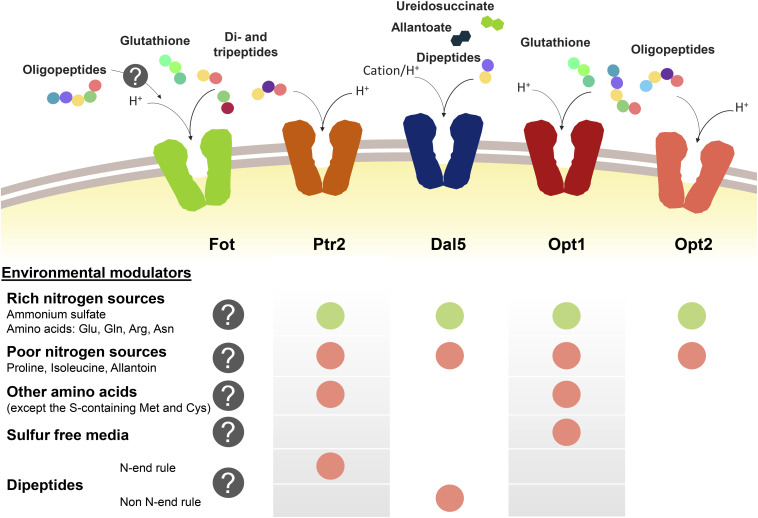
Oligopeptide transport systems in *S. cerevisiae* wine strains and the environmental modulators for their expression. Down-regulation is marked with a green circle and up-regulation is marked in red. The gray circle with the question mark indicates the lack of knowledge relating to Fot expression or consumption of oligopeptides longer than two or three amino acid residues.

Peptide transport in yeast cells greatly depends on the transport system and environmental factors, specially the presence of other nitrogen sources such as ammonium or amino acids (Figure 1). Nitrogen sources regulate peptide transport both at transcriptional level and by modulating the activity and degradation of enzymes and transporters. Ammonium and amino acids such as glutamate, glutamine or arginine are considered as rich nitrogen sources because they support a fast cell growth. However, the consideration as preferred nitrogen source also derives from the capacity of not de-repressing the uptake of alternative nitrogen sources held by the nitrogen catabolite repression (NCR) pathways (Cooper, 1982, 2002; Cooper and Sumrada, 1983; Basrai et al., 1992; Magasanik and Kaiser, 2002; Boer et al., 2007; Crépin et al., 2012). In *S. cerevisiae*, this repression by NCR affects *PTR2*, *DAL5*, and *OPT1-2*. In conditions of nitrogen starvation or presence of poor nitrogen sources such as allantoin or proline, the expression of *PTR2*, *OPT1-2*, and even *ASP3* is triggered (Bon et al., 1997; Lubkowitz et al., 1998; Magasanik and Kaiser, 2002). Although permease Dal5 is expressed constitutively for allantoate transport, it is also affected by NCR in the same way as Ptr2 and OPT transporters (Rai et al., 1987). For Ptr2, even micromolar concentrations of the non-preferred amino acids overcome NCR-mediated repression by ammonium, acting as inducers of peptide assimilation (Island et al., 1987; Perry et al., 1994). Expression of *OPT1* is also de-repressed by most non-preferred amino acids, particularly by leucine and tryptophan, however, *OPT1* is specifically induced in sulfur starvation conditions (Basrai et al., 1992; Wiles et al., 2006a). Therefore, methionine and cysteine do not belong to the group of amino acids inducers of *OPT1*. On the other hand, the expression of *OPT2* seems not to be affected by amino acid concentrations in the same way as *OPT1* or *PTR2* (Wiles et al., 2006a).

Another regulatory element of peptide transport is the amino acid sensor complex Ssy1p-Ptr3p-Ssy5p (SPS). Due to its sensor activity, SPS regulates the expression of several amino acid permeases depending on the presence of the corresponding substrate in the medium (Forsberg and Ljungdahl, 2001). In the same way, SPS is involved in the amino acid-driven regulation of Ptr2 and Opt1 expression and it has been demonstrated that the presence of *SSY1* and *PTR3* is required for the induction of both *PTR2* and *OPT1* (Didion et al., 1998; Wiles et al., 2006a).

Additionally, the import of dipeptides with basic and bulky amino acids in the N-terminal position (N-end rule dipeptides) upregulates the *PTR2* expression. This group of dipeptides bind to Ubr1p and activate the degradation of Cup9p, a transcriptional repressor of *PTR2* (Byrd et al., 1998; Turner et al., 2000; Hauser et al., 2001; Du et al., 2002). This *PTR2* upregulation mechanism initiates a positive feedback loop for the preferential uptake of N-end rule peptides by Ptr2 (Homann et al., 2005). Cup9p also represses the expression of *OPT2*, although it is unknown whether the N-end rule dipeptides have the same inducing effect as for *PTR2*. On the other hand, *OPT1* seems not to be affected by Cup9p. Contrary to the effect on *PTR2* or *OPT2*, Cup9p upregulates the expression of *DAL5* (Wiles et al., 2006a; Cai et al., 2007).

Fungal Oligopeptide Transporters expression has not been thoroughly studied yet. However, it has been shown that wine strains containing Fot1 and Fot2 initially consume small peptides from grape must before using some amino acids, such as histidine, alanine or glutamine, indicating that oligopeptides are a preferred source of nitrogen during the early stages of fermentation. This suggests that *FOT* genes may be expressed in *S. cerevisiae* wine strains from the beginning of fermentation (Marsit et al., 2016). However, analyzing the expression of *FOT* genes with different nitrogen supply in the medium would be helpful to better define the physiological role of these transporters throughout wine fermentation.

The divergent but complementary regulation in the expression of the peptide transport systems allows yeast to adapt to various environmental conditions and nutrient availabilities. However, Homann et al. (2005) suggested that oligopeptide transport seems to be subject to conflicting evolutionary pressures. The peptide transport machinery not only transports peptides serving as nutrients, but also antimicrobial peptides, which makes them a source of vulnerability for the cell. This assumption is based on the reported import of nikkomycin by di- and tripeptide transporters in pathogenic *C. albicans*, which initiated the development of peptide-analog antifungal drugs (McCarthy et al., 1985; St Georgiev, 2000). This idea is therefore only formulated from a clinical context perspective; theorizing about oligopeptide transporters as a source of vulnerability for yeasts would require further investigation of the hypothetic impact of antifungal peptides import by POT, OPT, FOT or the other permeases on yeast adaptation to natural, fermentative environments.

## Role of Oligopeptide Consumption in the Adaptation of *S. Cerevisiae* to the Wine Environment

Wine fermentation conditions constitute a harsh and stressful environment, exerting a great selective pressure on yeasts present in grape must. Actually, there are several sources of stress in grape must: the osmotic pressure caused by the high concentrations of sugar, high acidity, presence of sulfites or oxidative stress. Other sources of stress emerge as a consequence of the progress of fermentation, such as nutrient depletion and ethanol production. Indeed, nitrogen content is a limiting factor for yeast growth: after the latency phase, yeasts grow exponentially until reaching a maximum population and fermentation rate, consuming most nitrogen sources; at the stationary phase, undergoing nutrient starvation, yeasts stop growing and consume sugars through fermentation, which progressively increases ethanol level, another stress factor. Therefore, in addition to its nutrient-linked properties, nitrogen sources and availability can also qualitatively affect various outcomes of alcoholic fermentation (Agenbach, 1977; Cramer et al., 2002).

*Saccharomyces cerevisiae* is the yeast species best adapted to the winemaking environment, maintaining over 90% viability during the stationary phase until complete sugar supply depletion (Marsit and Dequin, 2015). Different molecular mechanisms have been shown to contribute to yeast adaptation to wine environments. Among them are gene copy number variations, chromosomal rearrangements, hybridization, introgression or horizontal gene transfer (Bidenne et al., 1992; Goto-Yamamoto et al., 1998; Rachidi et al., 1999; Pérez-Ortín et al., 2002; Dunn et al., 2005, 2012; Belloch et al., 2009; Novo et al., 2009; Stambuk et al., 2009; Esberg et al., 2011; Libkind et al., 2011; League et al., 2012; Morales and Dujon, 2012; Zimmer et al., 2014; Marsit et al., 2015, 2017; Legras et al., 2018).

Novo et al. (2009) sequenced the genome of *S. cerevisiae* wine strain EC1118 to investigate the molecular mechanisms that participate in the adaptation of wine yeasts to their environment. Sequence comparisons with other *S. cerevisiae* genomes revealed three genomic regions, A, B, and C, that had been acquired by HGT. Regions B and C had been shown to originate from two distantly related yeast species also found in wine, *Zygosaccharomyces bailii* and *T. microellipsoides*, respectively (Novo et al., 2009; Marsit et al., 2015). The three regions contained 39 putative genes coding for proteins involved in carbon and nitrogen metabolism, with *FOT* genes in region C among them. Region C was widespread among *S. cerevisiae* wine strains, albeit showing different rearrangements and/or gene losses. However, the two tandem genes *FOT1-2* encoding peptide transporters were strongly conserved in all the wine strains carrying region C. Some of the strains only conserved one copy of these genes, evolving into a new isoform of the *FOT* family with a high degree of homology, *FOT3* (Tables 2A,B). This result supported the idea that FOT transporters could confer an adaptive advantage within the wine environment (Marsit et al., 2015). To demonstrate this hypothesis, Marsit et al. (2015) evaluated wine strain 59A containing *FOT1-2* and the corresponding fot1-2-deleted mutant in fermentation experiments. Natural grape must was used because of its diverse sources of nitrogen that include oligopeptides, enabling the assessment of the strains into the complex matrix usually colonized by wine strains. Although the wild-type strain 59A showed lower fermentation and growth rates during the first hours of fermentation, mutants for fot1-2 eventually had a 12% lower biomass and a higher mortality rate than 59A, leading to residual fructose at the end of fermentation, an indicator of incomplete fermentation (Marsit et al., 2015). Since Fot1 and Fot2 are responsible for a wider range of oligopeptide transport (Damon et al., 2011), the assumption was that the greater consumption of oligopeptides by FOT not only increased biomass but also improved cell viability and fermentation performance in *S. cerevisiae*. Additionally, competition experiments between the wild-type and fot1-2 mutants on natural grape must revealed the competitive advantage of the wild-type over the mutant, demonstrating the beneficial role of Fot1-2 transporters in the adaptation of *S. cerevisiae* to the wine environment (Marsit et al., 2015).

**FIGURE 2:**
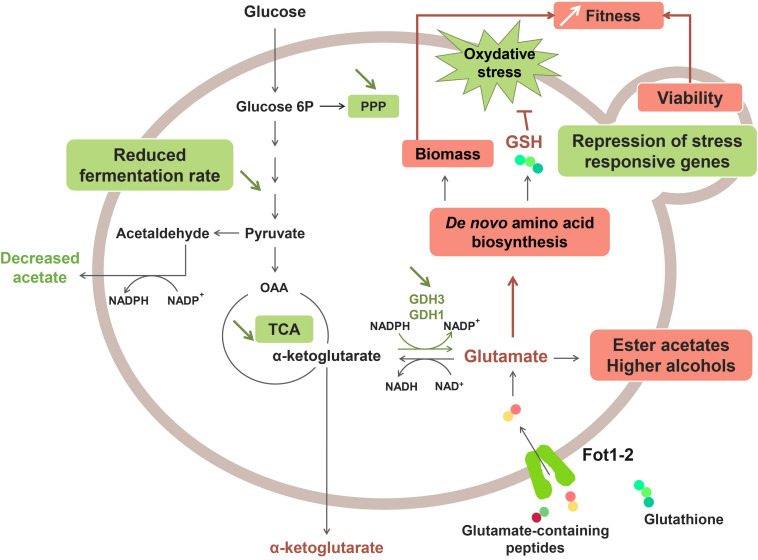
Effect of Fot1-2-mediated peptide uptake on the metabolism of 59A in oenological conditions. Molecules with higher production and upregulated pathways are marked in red, whereas downregulation and lower production are marked in green.

A recent study revealed that the greater consumption of glutathione and glutamate-rich peptides by Fot1-2 entailed a remodeling of the central carbon and nitrogen metabolism pathways (Marsit et al., 2016; Figure 2). Transcriptomic analyses showed that several genes involved in glutamate production (*GDH1* and *GDH3*), glycolysis, the tricarboxylic acid (TCA) cycle and the pentose-phosphate pathway (PPP) were downregulated in strain 59A (Marsit et al., 2016). The assumption is that the reverse conversion of α-ketoglutarate into glutamate is repressed due to high cell concentrations in glutamate originating from the consumption of glutamate/glutamine-rich oligopeptides. Consequently, α-ketoglutarate levels increase, leading to the repression of the upstream catabolic pathways, that is, TCA and glycolysis. Likewise, the downregulation of genes involved in the glycolytic pathway would explain the lower fermentation rate that was previously observed for 59A in comparison with the fot1-2 deleted strain. Furthermore, the NADP^+^/NADPH balance of the cell was also affected by the repression of α-ketoglutarate conversion into glutamate, resulting in a lower consumption of NADPH. NADPH accumulation agrees with the lower levels of acetate observed for 59A, since acetate production is an important source of NADPH. Additionally, the higher availability of glutamate derived from peptides acts as a nitrogen signal in the cell, inducing genes involved in the *de novo* biosynthesis of amino acids and glutathione. Higher concentrations of glutathione and NADPH have a positive impact on the protection against oxidative stress, which correlates with the repression observed for genes involved in the oxidative stress response (Marsit et al., 2016). Therefore, the assumption is that FOT contribute to the better response of 59A against oxidative stress, which is in accordance with its better survival at the end of fermentation when compared with fot1-2 deleted strains (Marsit et al., 2015). Furthermore, an accumulation of α-keto acids as a result of higher nitrogen availability was observed, leading to the increased production of ester acetates and fusel alcohols (Hazelwood et al., 2008; Rollero et al., 2015; Marsit et al., 2016) that are volatile molecules responsible for fruity and floral aromas in wine. Although high concentrations of fusel alcohols can have a detrimental effect on wine aroma, the increased levels of these molecules in wines fermented with 59A remained below 300 mg/L; at this concentration, it has been reported that fusel alcohols add a desirable level of complexity to wine aroma (Rapp and Versini, 1991; reviewed by Swiegers et al., 2005). These results, along with the decrease in acetate production, imply an improvement on the aroma profile of wines that is developed during fermentation. Consequently, a greater consumption of oligopeptides from grape must led by FOT constitutes a desirable oenological trait in yeasts. While that improvement of fermentation kinetics associated to biomass formation and cell survival is probably the main selection pressure permitting wine yeast strains adaptation, these potentially beneficial organoleptic properties, together with the reported positive impact on fermentation efficiency and cell viability, suggest a possible influence of domestication in Fot acquisition by *S. cerevisiae* wine strains. Humans, in their eagerness to obtain aromatically attractive wines and enhance the fermentation process, may have indirectly chosen those yeast inocula with strains containing Fot, i.e., strains able to consume more peptides from grape musts.

Currently FOT are the only oligopeptide transporters whose impact in the winemaking context has been evaluated. In other ecological niches, such as nectar, a recent study on the role of nitrogen as a competitive resource has shown that nitrogen scavenging genes, including oligopeptide transporters, drive priority effects on the microbial communities (Dhami et al., 2016). For *C. albicans*, PTR and OPT transporters have been shown to play a crucial role in nutrient acquisition, contributing to the success of the species as a commensal or pathogenic organism in mammalian hosts (Dunkel et al., 2013). In addition to the strong evidences provided for FOT supporting the crucial role of oligopeptide consumption, these results open new prospects for studying in more depth the other families of peptide transporters during wine fermentation.

## Conclusion and Prospects

Oligopeptides present in natural ecosystems constitute a non-negligible nitrogen source for yeasts. Despite the substantial advances recently made on the important role of oligopeptides during winemaking, many pending questions remain to be thoroughly investigated. First, it would be necessary to improve the existing methodologies dedicated to the quantification and qualitative analysis of peptide fractions within foods and beverages. Despite some previous work on this issue (Yokotsuka et al., 1975; Dale and Young, 1989; Monteiro et al., 2001; Moreno-Arribas et al., 2002; De Person et al., 2004; Takahashi et al., 2012), there is currently no standardized, thorough yet less laborious protocols that could be adapted to any sample with complex protein fractions. With a better knowledge of oligopeptide composition in grape musts, it would be easier to mimic oligopeptide matrices in the synthetic media normally used in a laboratory, allowing the development of new research subjects. For example, the influence of nitrogen availability on gene expression has been thoroughly studied in winemaking conditions, although none of these studies ever considered oligopeptides as potential modulators of gene expression (reviewed by Gobert et al., 2019). Oligopeptides have also been omitted from other studies concerning the ability of *S. cerevisiae* and non-*Saccharomyces* species to consume different nitrogen sources during winemaking fermentation (Crépin et al., 2012; Cubillos et al., 2017; Gobert et al., 2017; Seguinot et al., 2018; Su et al., 2020), or the effect of different nitrogen conditions in the adaptation of *S. cerevisiae* to wine environment (Ibstedt et al., 2015; Brice et al., 2018). This lack of information, contrasted with previous results on the adaptive advantage conferred by a greater consumption of oligopeptides due to FOT (Marsit et al., 2015, 2016), suggest a very promising field of study. Furthermore, analysis of peptide matrices would contribute to the characterization of oligopeptide transporters’ substrate specificities. A better characterization of transporters’ oligopeptide preferences in yeast definitely is a matter of interest not only for fermentation technologies, but also in distinct fields such as nutrition or drug design (Nielsen and Brodin, 2003; Ito et al., 2013). Finally, studying FOT expression is still required to refine the description of the peptide transport regulation already provided by the analysis of Ptr2, Opt1, Opt2, and Dal5. Understanding the physiological role of the novel FOT family in wine-related yeasts would help elucidate peptide catabolism and its interactions with other metabolic pathways, unraveling potential new functions of peptides within yeast life cycle and its relation to wine environment.

## Author Contributions

CB-R and VG drafted the manuscript. CB-R wrote the manuscript. All authors created the tables and figures, read and approved the final manuscript. SM and VG provided the writing guidance and revised the manuscript.

## Conflict of Interest

The authors declare that the research was conducted in the absence of any commercial or financial relationships that could be construed as a potential conflict of interest.

## Footnotes

1 http://aps.unmc.edu/AP/main.php

2 http://www.tcdb.org
